# Correlation Between Aneurysm Dome Direction and Patient Outcomes Following Surgery for Ruptured Anterior Communicating Artery Aneurysms

**DOI:** 10.7759/cureus.48051

**Published:** 2023-10-31

**Authors:** Ashis Patnaik, Jaidev S, Arunkumar Sekar, Sumit Bansal, Rabi N Sahu

**Affiliations:** 1 Department of Neurosurgery, All India Institute of Medical Sciences, Bhubaneswar, Bhubaneswar, IND

**Keywords:** aneurysms, ruptured anterior communicating artery, patient outcome surgery, aneurysm, direction of dome

## Abstract

Background

Anterior communicating (Acom) artery aneurysms are the most common type of intracranial aneurysms. Despite the enormous advancements in the field of endovascular surgery for intracranial aneurysms, open surgical clipping of aneurysms remains the most durable management of Acom aneurysms. There have been various studies based on the clinical-radiological profile and outcome of open clipping for intracranial aneurysms, but the correlation of the direction of the aneurysm with the surgical outcome remains incompletely defined.

Aim

To analyze the correlation between the direction of the aneurysm dome and patient outcomes after surgery for ruptured Acom artery aneurysms.

Materials and methods

CT angiography of the brain was done in all patients pre-operatively as part of the standard treatment protocol. Retrospective data was collected from an inter-departmental computerized database, which included the patient’s details, history, investigations, a course in the hospital from admission to discharge, and an operative note by the surgeon. Prospective data was collected from patients with ruptured Acom artery aneurysms. We analyzed the relationship between the direction of the dome of the Acom aneurysm and preoperative, intraoperative, and postoperative variables.

Results

Of the 48 patients who underwent surgery for ruptured aneurysms, 34 (70.8%) were female and 14 (29.2%) were male. Among the 27 cases with anteriorly projecting aneurysms, 11 (40.7%) experienced postoperative complications. Of the five cases with posteriorly projecting aneurysms, two (40%) experienced postoperative complications. One-third of cases of the inferior dome direction and 10% of cases of the superior direction of the dome of aneurysm were also found to have postoperative complications. However, using the chi-square test, the association between postoperative complications and the direction of the aneurysm was determined to be statistically non-significant (p-value = 0.93).

Conclusion

The findings of our investigation indicate that aneurysms with superior projection exhibit the lowest likelihood of experiencing intraoperative rupture. However, it was seen that the outcome was influenced by dome projection throughout the three-month follow-up. The findings of our study indicate that aneurysms with a superior orientation exhibit the least likelihood of experiencing intraoperative rupture.

## Introduction

The most frequent type of intracranial aneurysm is an anterior communicating (Acom) artery aneurysm, which accounts for up to 34% of all intracranial aneurysms [1, 2]. Ruptured Acom aneurysms pose exceptional surgical challenges due to their close proximity to the optic system and the varied morphology of the Acom artery, anterior cerebral artery, and its perforators. This complexity makes surgical intervention for Acom aneurysms more difficult and susceptible to intraoperative and postoperative complications compared to aneurysms in other locations [3, 4]. Moreover, compared to aneurysms in other places, Acom aneurysms appear more likely to rupture during surgery. Even at diameters that are deemed adequate for prudent treatment of aneurysms in other places, Acom aneurysms are nonetheless more likely to rupture [5, 6]. There have been several studies relying on the clinical-radiological characteristics and results of open surgical clipping for cerebral aneurysms. However, the relationship between the aneurysm's dome's direction and the surgical result is still not fully understood [7, 8]. The available literature provides some insight into how the direction of the dome affects choosing the approach side, the probability of perforator injury, and the Glasgow outcome rating [9, 10]. However, additional factors like the impact of the aneurysm's direction on intraoperative blood loss, the length of the procedure, the length of the hospital stay, and postoperative problems have not been fully investigated [11]. We try to close this information gap with this study. The results of this study will probably affect how decisions are made and what treatments are available for bursting Acom aneurysms. On this subject, relatively few studies have been conducted in India, and none have come from the East. This study investigated the correlation between the orientation of the aneurysm dome and the postoperative outcomes of patients who underwent surgery for ruptured Acom aneurysms.

## Materials and methods

The study was conducted in the Department of Neurosurgery at AIIMS Bhubaneswar over a span of 1.5 years, from January 2019 to January 2022. It was both a retrospective and prospective observational study. During this period, patients were enrolled and observed for a duration after inclusion in the study. The data of patients operated on for Acom aneurysms from August 2017 to January 2019 were retrieved from medical record systems and were analyzed retrospectively. For the prospective group, consent was obtained at the time of enrollment. For the retrospective analysis, consent was secured during subsequent follow-up visits. Thus, this study encompasses data analyzed both prospectively and retrospectively. All patients who met the inclusion criteria and underwent surgery in the Department of Neurosurgery at AIIMS Bhubaneswar between August 2017 and January 2022 were included. Inclusion criteria include patients with ruptured Acom artery aneurysms who have undergone surgery in our institute from August 2017 until January 2022. Exclusion criteria included patients with any aneurysms other than Acom aneurysms, those with multiple aneurysms, and patients suffering from connective tissue disorders or other systemic diseases, such as polycystic kidney disease, which are associated with a high prevalence of concomitant intracranial aneurysms.

Data collection tools

CT angiography of the brain was done in all patients pre-operatively, which is part of the standard treatment protocol. Retrospective data was collected from a well-maintained interdepartmental computerized database, which included the patient's details, history, investigations, course in the hospital from admission to discharge, and an operative note by the surgeon. Prospective data was collected from patients with ruptured Acom artery aneurysms presenting to All Indian Institute of Medical Sciences (AIIMS) Bhubaneswar. Computerized and offline data (patient's history, radiological imaging, preoperative workup, intra-operative course, and postoperative course) were maintained. The data thus collected was analyzed statistically. Confidentiality with regard to patient data was maintained in both retrospective and prospective samples. This is an observational study. There were no complications or untoward events for the patient because of the study. Complications, a part of the natural history of the disease, were managed by the neurosurgical team of AIIMS Bhubaneswar as per the standard treatment protocol. The study followed the Indian Council of Medical Research (ICMR) and Good Clinical Practice (GCP) guidelines. It commenced only after clearance from the Institutional Ethics Committee (IEC) with IRB number IEC/AIIMS BBSR/PG Thesis/2020-21/09. We analyzed the relationship between the direction of the dome of the Acom aneurysm and the following at the presentation for the World Federation of Neurological Surgeons (WFNS) grade.

Furthermore, we assessed intraoperative outcomes, taking into account factors like the duration of the surgery, the incidence of intraoperative rupture, the amount of blood loss, and the surgeon's comfort. Surgeon comfort relates to the easiness the surgeon finds in clipping the aneurysm. It is purely a subjective finding. Notably, this measure of surgeon comfort was only evaluated in the cohort of patients who were analyzed prospectively.

We evaluated various postoperative outcomes, including Glasgow outcome score at discharge, modified rank-in score at discharge, Glasgow outcome score at 3-month follow-up after discharge (only for prospective samples), modified rank-in score at 3-month follow-up post-discharge (only for prospective samples). Additionally, we examined the onset of new neurological deficits, the duration of the patient's hospital stay, their length of stay in the ICU, the duration they were on a ventilator, and the necessity for a second surgery.

Postoperative complications checked were seizures, vasospasm, hydrocephalus, and meningitis. During the surgical procedure, the angle of the aneurysm's dome projection was carefully observed. This observation was later confirmed by comparing it to preoperative CT angiography and 3D reconstruction. The plane of the planum sphenoidale is taken as the horizontal plane or zero-degree plane. Four quadrants were defined in relation to this plane: anterior (+45° to -45°), posterior (135° to -135° (or 225°)), superior (+45° to +135°), and inferior (135° to -45°). Any new-onset motor weakness, worsening of pre-existing weakness, development of aphasia, or falling Glasgow Coma Scale (GCS) were considered new-onset neurological deficits. In our institute, the standard surgical steps for clipping the Acom aneurysm begin with the pterional approach, which we have employed for all cases. A semicoronal cut is made, starting from the upper zygomatic edge and extending to the medial aspect of the forehead, beginning 5mm anterior to the tragus.

On the face of the frontal region of the Sylvian veins, the Sylvian fissure is divided. The spatula is introduced to grasp the frontal lobe once the Sylvian fissure has been opened, and it is then gradually pulled back towards the chiasma. The internal carotid artery (ICA) is stabilized before the membrane of the arachnoid is cut, exposing the opposing optic nerve. The optic chiasma's anterior portion is then separated. Once beyond the lower terrain of the Acom artery, the opposite side A1, is fixed. The next step involves accessing the aneurysmal peduncle. Before proceeding with the neck clipping of the aneurysm, it is crucial to establish five blood vessels: the symmetrical A1, A2, and Acom arteries. We developed a "surgeon comfort" score, a straightforward, objective scoring system that enables us to compare and evaluate surgical procedures' ease and difficulty from an objective standpoint. The data were analyzed through the computer software tool SPSS 21.0 after thorough data cleaning. Categorical variables were expressed in terms of proportion or percentage. A Chi-square test was applied to find out if there was any association between these variables. Continuous variables were presented in terms of mean ± SD. Ab=n ANOVA test was applied to find out any significant difference between the means of these variables.

## Results

Of the 48 patients with ruptured Acom-aneurysms who underwent surgery, 34 (70.8%) were females, and 14 (29.2%) were males. Among a total of 48 ruptured Acom-aneurysms cases posted for surgery, a maximum of 20 patients (41.7%) belonged to the 51-60 years age group. Thirteen patients (27.1%) belonged to 61-70 years of age group. Only one patient (2%) belonged to the <30 years age group. Among a total of 48 ruptured Acom-aneurysms cases, a maximum of 27 patients (56.3%) had the anterior direction of the dome. A total of 10 patients (20.8%) had superior direction of the dome. Only six (12.5%) and five (10.4%) patients had inferior and posterior directions of the dome, respectively. 

Table 1 describes the quantity of blood loss during surgery according to the dome of ruptured Acom-aneurysms.

**Table 1:** Blood loss in relation to the direction of the Acom-aneurysm dome. ml: Millimeters; Acom: Anterior communicating.

Directions of Aneurysm	Minimum Blood Loss in ml	Maximum Blood Loss in ml	Mean blood loss in ml	SD	One-way ANOVA	
Anterior	100	800	455.56	159.5	F=1.069, p=0.37	Brown–Forsythe test =0.96 P=0.43
Posterior	200	600	370	156.5		
Superior	200	1200	525	292.7		
Inferior	150	600	375	175.3		

There was a mean blood loss of 525 ml in the superior direction and a mean blood loss of 370 ml in the posterior direction. Anterior direction had a mean blood loss of 455.5 ml. The inferior direction had a similar mean blood loss (375 ml) in the posterior direction. However, the difference between these means and their variations were found to be statistically non-significant by applying the one-way ANOVA test. Table 2 describes the duration of surgery according to the dome of ruptured Acom-aneurysms.

**Table 2:** Duration of surgery in relation to the direction of the Acom aneurysm dome. Acom: Anterior communicating.

Directions of Aneurysm	Minimum Surgery Duration in Minutes	Maximum Surgery Duration in Minutes	Mean Duration of Surgery in Minutes	SD	One-way ANOVA	
Anterior	120	300	207.78	48.4	F=0.313p=0.816	Brown–Forsythe test = 0.239; P=0.867
Posterior	150	360	222	83.7		
Superior	150	240	198	32.2		
Inferior	180	270	215	39.8		

A maximum of 222 minutes were consumed by the surgeon for the posterior direction. The inferior direction consumed a similar mean figure of 215 minutes. The surgeon needed comparatively lesser mean duration of surgery for anterior (207.78 minutes) and superior (198 minutes) directions, respectively. However, the difference between these means, as well as their variations, were found to be statistically non-significant by applying one one-way ANOVA test. Intra-operative ruptures were reported in five cases, each from the inferior (83.3%) and two cases from the posterior (40%) direction of aneurysms. Only one case of intraoperative rupture (3.7%) was reported for anterior direction. There was no case of intra-operative rupture reported in the superior direction. Temporary clipping was done in all cases equally. The association between these intraoperative ruptures and the direction of the dome of the aneurysm was found to be statistically highly significant (p-value:<0.01) by applying the Chi-square test. Table 3 describes the surgeon's comfort rating according to the dome of ruptured Acom-aneurysms.

**Table 3:** Surgeon's comfort rating in relation to the direction of the Acom aneurysm dome. Acom: Anterior communicating.

Directions of Aneurysm	Mode of Surgeon Comfort	Mean Surgeon Comfort	SD	One-way ANOVA
Anterior	1	1.63	0.74	F=1.07p=0.39
Posterior	1	1.0	0.0	
Superior	1	2.0	0.36	
Inferior	2	2.33	0.33	

It is to be noted that only those cases who have been posted for surgery (18 cases) during the study period could provide this data. Clinical records of old cases (30 cases) could not provide the data for surgeon comfort level. The mean surgeon comfort level is highest (2.33) in the case of the inferior direction of the dome and lowest (1.0) in the case of the posterior direction of the dome. However, this ANOVA was found to be statistically non-significant by applying the one-way ANOVA test. Table 4 depicts the GOS at discharge and GOS at three months after discharge with respect to the dome of ruptured Acom-aneurysms.

**Table 4:** Comparison of GOS based on the direction of the aneurysm dome. GOS: Glasgow Outcome Score.

Directions of Aneurysm	Mean GOS at Discharge (n=48)	SD	One-way ANOVA	Mean GOS After Three Months of Discharge (n=18)	SD	One-way ANOVA
Anterior	4.2	1.4	F=5.67; p=0.002	3.38	1.8	F=1.6; p=0.23
Posterior	1.6	0.9		1.0	0.0	
Superior	4.4	1.0		4.33	1.2	
Inferior	3.8	1.6		4.33	1.1	

This is to be noted that only those cases who have been posted for surgery (18 cases) during the study period, could provide the data for the GOS after three months of discharge. Clinical records of old cases (30 cases) could not provide the data for the same. Mean GOS at discharge are higher in the case of superior (4.4) and anterior (4.2) directions of the dome and lowest (1.6) in the case of the posterior direction of the dome. The ANOVA was found to be statistically highly significant by applying a one-way ANOVA test. Mean GOS after three months of discharge is highest (4.33) in the case of both superior and inferior directions of the dome and lowest (1.0) in the case of the posterior direction of the dome. However, this ANOVA was found to be statistically non-significant by applying the one-way ANOVA test. Table 5 depicts the modified Rankin Scale (mRS) at discharge and mRS at three months after discharge with respect to the dome of ruptured Acom-aneurysms.

**Table 5:** Comparison of modified Rankin scale based on the direction of the aneurysm dome. mRS: Modified Rankin scale.

Directions of Aneurysm	Mean mRS at the Time of Discharge (n=48)	SD	One-way ANOVA	Mean mRS after Three Months of Discharge (n=18)	SD	One-way ANOVA
Anterior	2.75	2.2	F=1.2; p=0.35	2.78	2.1	F=0.29; p=0.82
Posterior	6.0	0.0		2.8	2.5	
Superior	2.0	1.5		2.1	1.6	
Inferior	2.67	2.1		2.8	2.1	

It is to be noted that only those cases who have been posted for surgery (18 cases) during the study period could provide the data for mRS after three months of discharge. Clinical records of old cases (30 cases) could not provide the data for the same. Mean mRS at discharge is highest (6.0) in the case of the posterior direction of the dome and lowest (2.0) in the case of the superior direction of the dome. Mean mRS after three months of discharge is almost similar (~score of 2.8) in the case of anterior, posterior, and inferior directions of the dome and lowest (2.1) in the case of the superior direction of the dome. However, this ANOVA was found to be statistically non-significant in both cases by applying a one-way ANOVA test. Table 6 describes the duration of hospital stay, ICU stay, and on-ventilator duration with respect to the dome of ruptured Acom-aneurysms.

**Table 6:** Duration of hospital stay and need for critical care based on the direction of the aneurysm dome.

Directions of Aneurysm	Mean Duration of Stay in Hospital in Days	SD	One-way ANOVA	Mean Duration of Stay in ICU in Days	SD	One-way ANOVA	Mean Duration of Stay on Ventilator in Days	SD	One-way ANOVA
Anterior	17.7	12.4	F=0.53; p=0.66	9.37	8.8	F=0.34; p=0.79	6.04	6.6	F=0.33; p=0.79
Posterior	14.2	9.8		10.6	9.5		5.6	6.1	
Superior	16.5	9.5		9.2	11.5		5.6	8.5	
Inferior	11.8	5.1		5.7	2.8		3.0	1.8	

The mean duration of hospital stay is highest (17.7 days) in the case of the anterior direction of the dome and lowest (11.8 days) in the case of the inferior direction of the dome. The mean duration of ICU stay is highest (10.6 days) in the case of the posterior direction of the dome and lowest (5.7 days) in the case of the inferior direction of the dome. The need for a ventilator was found to be of the longest duration (mean of 6.04 days) in the case of the anterior direction of the dome and was of the shortest duration (mean of 3.0 days) for the inferior direction of the dome. However, the ANOVA between these directions was found to be statistically non-significant in all three cases by applying a one-way ANOVA test (p=0.79). Twelve cases (44.4%) out of 27 cases needed a second surgery for the anterior direction of the dome of the aneurysm. Two cases (40%) out of five posterior directions of the dome of the aneurysm needed a second surgery. One-third of cases of six inferior directions of the dome and 10% of 10 superior directions of the dome of aneurysm did need second surgery. However, the association between the need for a second surgery and the direction of the dome of the aneurysm was found to be statistically non-significant (p-value-0.86) by applying a Chi-square test. Eleven cases (40.7%) out of 27 cases had postoperative complications for the anterior direction of the dome of the aneurysm. Two cases (40%) out of five posterior directions of the dome of aneurysms had some sort of postoperative complications. One-third of cases of six inferior directions of the dome and 10% of 10 superior directions of the aneurysm dome were also found to have postoperative complications. However, the association between the occurrence of postoperative complications and the direction of the aneurysm dome was found to be statistically non-significant (p-value-0.93) by applying the Chi-square test. All the major complications like hydrocephalus, seizures, vasospasm, meningitis, and IVH were reported in equal proportion (7.4% each) in the case of the anterior dome of the direction of the aneurysm. One case (20%) of the posterior direction of the dome aneurysm reported postoperative seizures. Similarly, only one case (16.6%) of the inferior dome of aneurysm was reported to have postoperative vasospasm. Among superior directions, two cases (20%) were reported to have hydrocephalus as a postoperative complication, and one case each (10%) was found to have seizures, vasospasm, and meningitis as postoperative complications. The association between the occurrence of these postoperative complications and the direction of the aneurysm dome was found to be statistically non-significant by applying a Chi-square test in each category. Table 7 describes the WFNS grading in relation to the direction of the dome of Acom-aneurysms.

**Table 7:** WFNS scores at presentation based on the direction of the dome of Acom-aneurysm. WFNS: World Federation of Neurological Societies.

Directions of Aneurysm	Mean WFNS Score at Presentation (n=48)	SD	One-way ANOVA
Anterior	2.7	1.2	F=3.69; p=0.019
Posterior	4	Nil	
Superior	2.2	0.91	
Inferior	2.17	0.98	

The mean WFNS score is highest (score of 4) with the posterior direction of the dome and lowest (score of 2.17) with the inferior direction. The ANOVA was found to be statistically significant (p-value=0.019) by applying a one-way ANOVA test.

## Discussion

In our study, among the total 48 ruptured Acom-aneurysm cases posted for surgery, 34 (70.8%) were female patients and 14 (29.2%) were male patients. Patient demographics of 300 Acom-aneurysm patients by Bohnstedt BN et al. [1] had similar gender distribution, with a higher incidence of Acom aneurysms in females than males. The Acom series by Ivan ME et al. also had a majority of females (64%) in their study [12]. Among the total 48 ruptured Acom-aneurysm cases posted for surgery, a maximum of 20 patients (41.7%) belonged to the 51-60 age group. This finding is consistent with the demographic findings in the study of Acom aneurysms by Bohnstedt BN et al. [1]. In the study by Ivan ME et al., the average age of the study population was 56 years [12]. The age of the patient has implications for patient outcomes. It is well known from previous studies that as the patient's age increases, the chances of a good surgical outcome decrease. On analyzing the percentage distribution of each of the four directions in the study population, it was noted that a maximum of 27 patients (56.3%) had an anterior direction of the dome. Ten patients (20.8%) had superior direction of the dome. Only six (12.5%) and five (10.4%) patients had inferior and posterior directions of the dome, respectively. These findings are consistent with previous studies. A study by Bohnstedt BN et al. [1] also showed that an anteriorly directed dome is the most common direction of the dome in aneurysms. The percentage distribution of various directions in their study is as follows: 48% anterior, 29% inferior, 17% superior, and 19% posterior [1]. Operative blood loss is a vital intraoperative parameter that has a bearing on patients' postoperative outcomes. In our study, there was a mean blood loss of 525 ml in the superior direction and a mean blood loss of 370 ml in the posterior direction. The anterior direction had a mean blood loss of 455 ml. The inferior direction had a mean blood loss (375 ml) similar to that of the posterior direction. However, the difference between these means, as well as their variations, were found to be statistically non-significant. On most occasions, intraoperative rupture of aneurysms or injury to perforator vessels is the primary source of bleeding. We have seen that in our study population, the superior direction is clinically significant for decreased risk of intra-op aneurysm rupture. However, the superior direction of the dome does not have the least blood loss. In our study, the superiorly directed dome had a relatively higher intraoperative blood loss than other directions. The higher blood loss in superiorly directed domes is probably because of the more extensive dissection of domes done in these cases. Posterior and inferior directions have a lower mean blood loss because less dissection of the dome is required in these cases. There are other studies available in the medical literature that have analyzed the relationship between intraoperative blood loss and the direction of the aneurysm [10-14]. When we analyzed the relationship between the intraoperative rupture of an aneurysm and the direction of the aneurysm, it was found that only one case of intraoperative rupture (3.7%) was reported for the anterior direction. There was no case of intra-operative rupture reported in aneurysms, with the dome pointing in a superior direction. Superiorly directed domes had a significantly lesser risk of intraoperative rupture than other directions. These findings are contrary to the findings in the study by Ivan ME et al. [12], where they found that quadrant dome projections did not enhance the probability of intraoperative rupture. Acom-aneurysm perforators are endangered by posteriorly projecting aneurysms during permanent clipping. These perforators are forced inferiorly into the triangle's lower corner by the aneurysm dome. Although it is simpler to visualize this corner than the upper corner, it is more dangerous when attempting to separate these perforators because of their tiny size and potential for dense adhesion to the aneurysm [13].

In their investigation, Sandalcioglu IE et al. [6] discovered that intraoperative aneurysm burst (IOR) does not affect the result. The death frequency after IOR rose, and the prognosis for IOR was insurmountable, which was validated by Bederson JB et al. [9]. We designed an objective scoring system to grade the technical ease and comfort level of the surgeon during the entire surgical procedure. We tried to analyze its relationship with the direction of the aneurysm. The mean surgeon comfort level is highest (2.33) in the inferior direction of the dome and lowest (1.0) in the posterior direction of the dome. However, the role of the direction of the aneurysm was insignificant in making the surgical procedure easy or difficult for the surgeon. Ivan ME et al. [12], in their research article on Acom-aneurysms, discovered that anterior as well as inferior extending aneurysms only affect the pre-communicating triangle, which is simpler for cutting microsurgically and had better results. Acom-aneurysms are more difficult to visualize, necessitate accessing the junctional triangle, and present surgical challenges due to superior and posterior dome extensions. The dome obscures the contralateral A2 section, making superiorly extending Acom-aneurysms the most difficult to detect [14]. When we evaluated the relation of the GOS with the direction of the dome of aneurysm, the mean GOS at discharge was higher in cases of superior (4.4) and anterior (4.2) directions of the dome and lowest (1.6) in cases of posterior direction of the dome. The ANOVA was found to be statistically significant. This is consistent with previous studies. The study by Ivan ME et al. [12] also had a similar outcome. In their study, they state that aneurysms directed superiorly and posteriorly have poor outcomes. The probable explanation for the worse outcome is that posteriorly projecting aneurysms hide Acom artery perforators. These perforators are forced downward into the lower corner of the communicating triangle by the aneurysm dome [13]. Because of their tiny size and potential for extensive adhesion to the aneurysm, these perforators are more dangerous to securely dissect. To maintain the blood flow through the hypothalamus as well as columns located in the fornix, which have an impact on endocrine as well as mental performance, respectively, perforator conservation is required [12]. Additionally, Debono B et al. [13] discovered that individuals with anteriorly extending Acom aneurysms had higher GOS scores. In the research conducted by Bohnstedt BN et al. [1], the mean GOS remained 4.6 at discharge and 4.8 at six-month and twelve-month follow-up, respectively, among patients with unruptured aneurysms. A total of 92% of patients had a good prognosis (GOS of 4 or 5), whereas only 8% had an unfavorable result (GOS of 3). The average GOS score among individuals with ruptured aneurysms was 3.8 at discharge, and it improved to 6.0 six months later and remained consistent four months and 12 months afterward. In their study, 67.5% of individuals who underwent microsurgical clip constriction following a subarachnoid hemorrhage from an Acom aneurysm achieved a favorable outcome, reflected by a GOS score of 4 or 5 [1]. This aligns with results from the previous International Trial, where 69.4% of all patients with subarachnoid hemorrhage who underwent microsurgical clipping reported a successful outcome one year post-treatment [14]. We analyzed the relationship between postoperative complications and the direction of the dome. Eleven cases (40.7%) out of 27 cases had postoperative complications in the anterior direction of the dome of the aneurysm. Two cases (40%) out of five posterior directions of aneurysms had some sort of postoperative complications. One-third of cases of the inferior direction of the dome and 10% of cases of the superior direction of the dome of aneurysm were also found to have postoperative complications. However, the association between the occurrence of postoperative complications and the direction of the aneurysm was found to be statistically non-significant. Our findings were consistent with the study findings by Bohnstedt BN et al. [1], in which, analyzing the extensions of the Acom-aneurysm dome, there were no statistically significant changes in results or comorbidities. Salary M et al. [11] investigated the effects of abrupt hydrocephalus in subarachnoid hemorrhage (SAH) patients and the results following shunting. They concluded that there was no meaningful advantage to shunting and that since shunted patients generally had worse conditions than non-operated patients, it was reasonable to assume that their outcomes would be poorer as well. We analyzed the relationship between WFNS scores at the presentation and the direction of the Acom aneurysm. The mean WFNS score is highest (score of 4) with the posterior direction of the dome and lowest (score of 2.17) with the inferior direction, which was found to be statistically significant. The probable explanation for our findings is that posteriorly directed domes at presentation do not present with a very thick SAH owing to their anatomical relation to the cisterns. Hence, they do not become symptomatic early and probably result in a delayed presentation. This delayed presentation may cause the worse WFNS score observed in the posteriorly directed dome of the aneurysm. Hunt and Hess score, Modified Fischer score, and WFNS score are three widely used clinical scoring systems for ruptured intracranial aneurysms [15].

Limitations

This study has several limitations. Firstly, the small sample size of the study is a drawback. Additionally, since the research was conducted at a single center, its relevance to a wider range of people is constrained. Our study included 18 prospective samples and 30 retrospective samples. Factors like surgeon comfort and three-month follow-up GOS/MRS scores could not be obtained for the retrospective group. In comparison, we managed a three-month follow-up for the prospective group. However, a longer follow-up would be necessary to understand the implications of the direction of the aneurysm for the long-term patient outcome. Taking all these limitations into consideration, a large prospective clinical study with long-term follow-up may be warranted to better understand the role of the direction of the Acom-aneurysm on patient outcomes.

## Conclusions

The results of our study show that patients with posteriorly directed domes of aneurysm tend to have a lower WFNS score at the time of presentation and the lowest GOS at the time of discharge. According to the study results, the direction of the aneurysm does not affect intraoperative blood loss, duration of surgery, duration of hospital stay, ICU stay, duration of stay on ventilator, need for second surgery, new-onset neurological deficit, or postoperative complications. This study shed some light on the role of the direction of the dome of aneurysms on various preoperative, intraoperative, and postoperative patient parameters. A larger prospective study with long-term follow-up is required to better understand the influence of the direction of the dome of Acom-aneurysm on patient outcomes.
